# Three-Dimensional Morphological Changes of the True Cleft under Passive Presurgical Orthopaedics in Unilateral Cleft Lip and Palate: A Retrospective Cohort Study

**DOI:** 10.3390/jcm9040962

**Published:** 2020-03-31

**Authors:** Prasad Nalabothu, Benito K. Benitez, Michel Dalstra, Carlalberta Verna, Andreas A. Mueller

**Affiliations:** 1 Department of Orthodontics and Pediatric Dentistry, University Center for Dentistry, 4031 Basel, Switzerland; prasad.nalabothu@unibas.ch (P.N.); michel.dalstra@dent.au.dk (M.D.); carlalberta.verna@unibas.ch (C.V.); 2 Department of Oral and Craniomaxillofacial Surgery, University Hospital Basel, 4031 Basel, Switzerland; benito.benitez@usb.ch

**Keywords:** cleft lip and palate, cleft palate, three-dimensional, presurgical orthopaedics, true cleft, passive plate, vomer

## Abstract

The aim of this cohort study was to quantify the morphological changes in the palatal cleft and true cleft areas with passive plate therapy using a new analysis method based on three-dimensional standardized reproducible landmarks. Forty-five casts of 15 consecutive patients with complete unilateral cleft lip and palate were laser scanned and investigated retrospectively. The landmarks and the coordinate system were defined, and the interrater and intrarater measurement errors were within 1.0 mm. The morphological changes of the cleft palate area after a period of 8 months of passive plate therapy without prior lip surgery are presented graphically. The median decrease in cleft width was 38.0% for the palatal cleft, whereas it was 44.5% for the true cleft. The width of the true and palatal cleft decreased significantly over a period of 8 months. The true cleft area decreased by 34.7% from a median of 185.4 mm^2^ (interquartile range, IQR = 151.5–220.1) to 121.1 mm^2^ (IQR = 100.2–144.6). The palatal cleft area decreased by 31.5% from a median of 334 mm^2^ (IQR = 294.9–349.8) to 228.8 mm^2^. The most important clinical considerations are the reproducibility and reliability of the anatomical points, as well as the associated morphological changes. We propose using the vomer edge to establish a validated measuring method for the width, area, and height of the true cleft.

## 1. Introduction

The area that surrounds the entrance from the oral into the nasal cavity in cleft lip and palate was described by Victor Veau as “fente vraie” [1], which we translate as “true cleft”. Whereas the curved vomer “portion du vomer incurvé” [1] together with the true cleft is referred to as “palatal cleft” and denotes the gap in palatal mucosa. All types of hard palate surgeries aim for tissue to cover the true cleft region in order to produce a functional seal between the oral and nasal cavities. Despite the fundamental clinical importance of the true cleft region in all cleft palate surgery techniques, to our knowledge this region has not previously been investigated in three dimensions.

The anatomical and functional alterations of the cleft lip and palate result in dimensional alterations in the palate. The exact morphology of these alterations in turn determines the extent of the necessary tissue shift at the time of surgical cleft closure and has consequences for healing and growth. The presurgical cleft palate morphology is therefore of great significance for the perioperative and long-term rehabilitation of patients.

However, there is no consensus on how to measure the cleft size and categorize its severity. This might be because the separation of the maxillary segments [2] is easier to measure, while the extent of tissue deficiency is difficult to quantify. Further, defining the landmarks and measuring the cleft palate are commonly performed in two dimensions [2,3,4,5], which represents a simplification of the three-dimensional (3D) complexity of the cleft palate. Moreover, there is a wide diversity of methodologies applied to describe the cleft palate morphology in children with complete unilateral cleft lip and palate. Some researchers measure only the separation between two segments anteriorly [4,5], whereas others measure the cleft width or area between the palatal shelves, which is commonly defined as the cleft area [3,6,7]. The cleft area is quantified as a percentage of the total palatal area rather than as an absolute number. The use of these methods to establish the correlation between the initial cleft size and outcome measurements, such as occlusion or midface skeletal development, has produced contradictory results due to ill-defined landmarks, low-quality dental casts, and lack of general reproducibility [7,8,9].

The previously used two-dimensional (2D) measurement techniques comprise direct measurements of real plaster casts and measurements of its photographs or photogrammetric models, and measurements of occlusal radiographs [2,3,4,5,6,7,10,11,12]. The projection of 3D points onto a 2D plane is affected by the orientation of the cast and the plaster-cast surface area. Most measurements in cleft lip and palate studies, such as of the inclination of the palatal shelves or the surface area of the palatal segments, are 3D in nature and therefore only strictly valid when evaluated in three dimensions [13,14].

The aim of this cohort study was to use a new analysis method based on 3D standardized, reproducible landmarks to quantify the morphological changes of the palatal cleft and true cleft areas under passive plate therapy.

## 2. Materials and Methods

### 2.1. Patients and Plaster Casts

This study retrospectively analysed 15 consecutive patients with complete unilateral clefts of the lip, alveolus, and palate who were treated at the last author’s (A.A.M.) institute. The subjects comprised 3 females and 12 males, and none of them had Simonart’s band. Each infant received passive plate therapy, that lead to 3 plaster casts that had been taken at the following different intervals (total of 45 casts): during the first week after birth (before passive plate therapy) (T0), 3–4 months after birth (ongoing passive plate therapy) (T1), and prior to primary surgery at around 8 months (at the end of passive plate therapy) (T2). Passive plate therapy was applied by the same surgeon (A.A.M.) to all patients. Informed consent was obtained from the children’s parents or guardians. The study was performed in accordance with the Declaration of Helsinki, and it was approved by the Ethics Commission of Northwest and Central Switzerland (EKNZ) (project-ID 2018-01561).

### 2.2. Passive Plate Therapy

After birth, an impression of the palate was taken in the awake infant using an individual impression tray and silicone (Epiform-flex, Dreve-Dentamid, Unna, Germany). The cleft depressions on the plaster cast were blocked out using soft putty (President, Coltène/Whaledent, Altstätten, Switzerland) in order to simulate a normal palatal vault shape and create a free space to the vomer mucosa, to the palatal shelves, and between the alveolar segments. The alternating application of monomer spray (Orthocryl liquid monomer, Dentaurum, Ispringen, Germany) and sprinkling of acrylic powder (Orthocryl polymethylmethacrylat, Dentaurum, Ispringen, Germany) formed a passive plate with a target thickness of 1.5–2.0 mm. A caregiver removed the plate once daily to clean and disinfect it (Octenisept, Schuelke, Norderstedt, Germany). A thin film of tasteless denture adhesive (Kukident Neutral Extra Strong, Kukident, Weinheim, Germany) kept the plate in place. The plate typically became unstable after 3–4 months, when it was renewed. The third impression was taken prior to the lip and palate repair in one cleft surgery at around 8 months.

### 2.3. Three-Dimensional Analysis

Plaster cast of the infants were scanned and digitized using a high-precision laser scanner (Iscan L2, Imetric Swiss 3D Scanning Systems, Switzerland, precision of <15 μm) and were exported in the STL (stereolithographic) file format. The exported models were then imported into dedicated 3D analysis software (Mimics version 20.0, Materialise, Leuven, Belgium).

**Figure 1:**
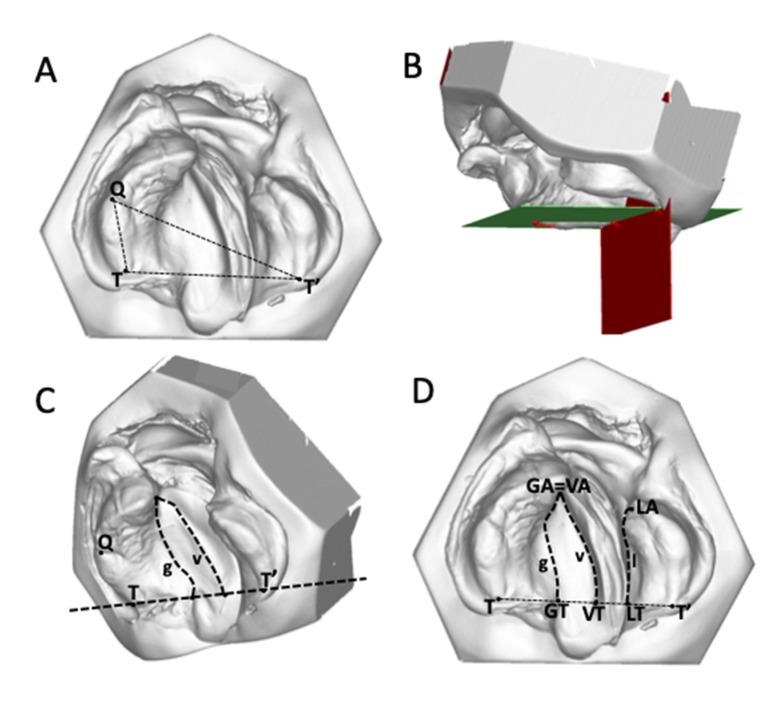
Establishment of a three-dimensional coordinate system. (**A**) and (**B**) A horizontal plane (green) is defined by (Q, T, T’), and a vertical plane (red) is defined by (T, T’). (**C**) and (**D**) The line between VT–VA denotes the vomer edge (v), the line between GT–GA denotes the palatal shelf ridge of the greater segment (g), and the line between LT–LA represents the palatal shelf ridge of the lesser segment (l).

**Figure 2:**
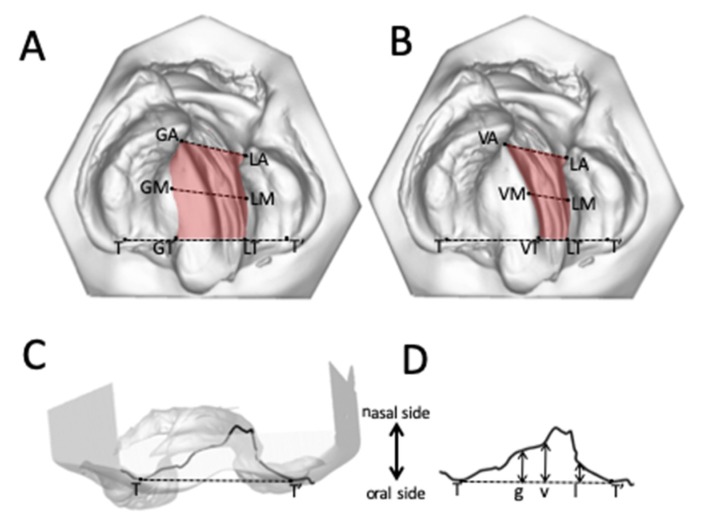
(**A**) Palatal cleft: palatal cleft width (dashed lines) and palatal cleft area (shaded area). (**B**) True cleft: true cleft width (dashed lines) and true cleft area (shaded area). (**C**) and (**D**) The height of the palate to the horizontal plane at the vomer edge (v) and at the greater (g) and lesser (l) palatal shelf ridges.

The 45 casts were analysed by marking 14 landmarks on each digitized model based on the principles of Stöckli [2] and Mazaheri et al. [10]. The 3D definitions of the measurement landmarks are presented in Table 1 and Table 2 and illustrated in Figure 1 and Figure 2.

The coordinate system was established as described by Botticelli et al. [15], by a horizontal plane passing through Q–T/T’ and a posterior vertical plane perpendicular to the previous one passing through T/T’. The most-anterior point on the palatal ridge of the greater segment (GA) and the most-anterior point of the vomer edge (VA) were at the same point (Figure 1C,D). This point is referred to anatomically as the innominate sulcus (or “unnamed furrow”) [16].

The palatal cleft was delimited by the greater segment’s palatal shelf ridge (g) at the junction to the vomer and the lesser segment’s shelf ridge (l). The true cleft was delimited by the vomer edge (v) and the lesser segment’s palatal shelf ridge (l). The height measurements were performed along the three paths g, l, and v at nine equidistant points each, generated from the ascending order of 0 to 100% (0%, 12.5%, 25%, 37.5%, 50%, 62.5%, 75%, 87.5%, 100%). The height in each point was measured perpendicular to the horizontal plane (Figure 2C,D). 

**Figure 3:**
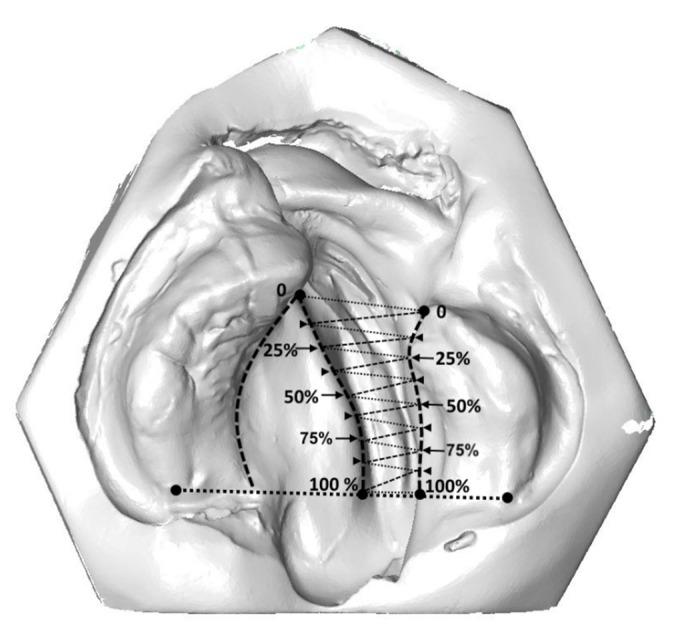
True cleft area measurement connecting equidistant points from the vomer edge to the lesser palatal shelf ridge (0% and 0%, 12.5% and 12.5%, and so forth) led to 8 equidistant quadrangles, which were split into two triangles each, leading to a total of 16 triangles. Surface measurements of defined areas were then approximated as the sum of its comprising triangles.

Connecting corresponding equidistant points from g to l and v to l (0% and 0%, 12.5% and 12.5%, and so forth) led to 8 equidistant quadrangles, which were split into two triangles each, leading to a total of 16 triangles (Figure 3). Surface measurements of defined areas were then approximated as the sum of its comprising triangles.

### 2.4. Statistical Analysis

The measurements made at time points T0 and T2 were compared using a Wilcoxon signed-ranks test. Statistical significance was assumed at *p* < 0.05. The abovementioned procedures for calculating the cleft width, cleft area, and height of the cleft edges were repeated for 15 of the 45 casts both by the same rater and by a second rater. The differences were investigated to quantify the measurement error of the method according to Dahlberg’s formula [17]. The statistical analysis was performed using Stata (version 15.1, StataCorp LLC, TX, USA).

## 3. Results

The analysis of landmark positioning in the 3D cast analysis showed that the intrarater measurement errors ranged from 0.7 to 0.9 mm and those for interrater measurements ranged from 0.5 to 1.0 mm.

### 3.1. Cleft Width

**Figure 4:**
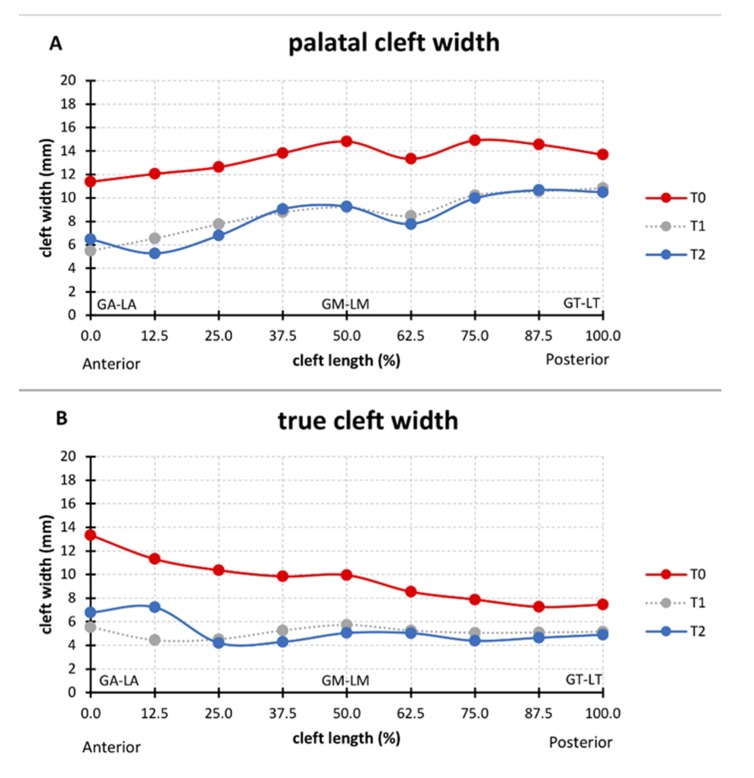
Median (**A**) palatal cleft width (PCW) and (**B**) true cleft width (TCW) before passive plate therapy (T0), after 3–4 month of passive plate therapy (T1), and prior to primary surgery at around 8 months (T2).

The median palatal cleft width at T0 was 11.4 mm (interquartile range, IQR = 9.8–14.4 mm) in the anterior region (GA–LA), 14.8 mm (IQR = 14.0–15.9 mm) in the midpalatal region (GM–LM), and 13.7 mm (IQR = 12.3–16.7 mm) in the posterior region (GT–LT). The median true cleft width was 13.3 mm (IQR = 10.6–14.4 mm) in the anterior region (VA–LA), 9.9 mm (IQR = 8.1–11.0 mm) in the midpalatal region (VM–LM), and 7.4 mm (IQR = 5.8–10.4 mm) in the posterior region (VT–LT). The narrowing of the palatal and true cleft from T0 to T2 resulted in its width becoming more even from anterior to posterior locations along the cleft (Figure 4). The median palatal cleft width decreased significantly at T2, from 11.4 to 6.5 mm (z = 3.237, *p* = 0.0012) in the anterior region (GA–LA), from 14.8 to 9.3 mm (z = 3.41, *p* = 0.0007) in the midpalatal region (GM–LM), and from 13.7 to 10.5 mm (z = 3.18, *p* = 0.0015) in the posterior region (GT–LT). Similar changes were seen in the true cleft width in the anterior region (VA–LA) (from 13.3 to 6.8 mm, z = 3.41, *p* = 0.0007), in the midpalatal region (VM–LM) (from 9.9 to 5.0 mm, z = 3.41, *p* = 0.0007), and in the posterior region (VT–LT) (from 7.4 to 4.9 mm, z = 3.24, *p* = 0.0012) (Figure 4). The median decrease (from T0 to T2) in cleft width was 38.0% for the palatal cleft, whereas it was 44.5% for the true cleft.

### 3.2. Changes in Palatal and True Cleft Areas

**Figure 5:**
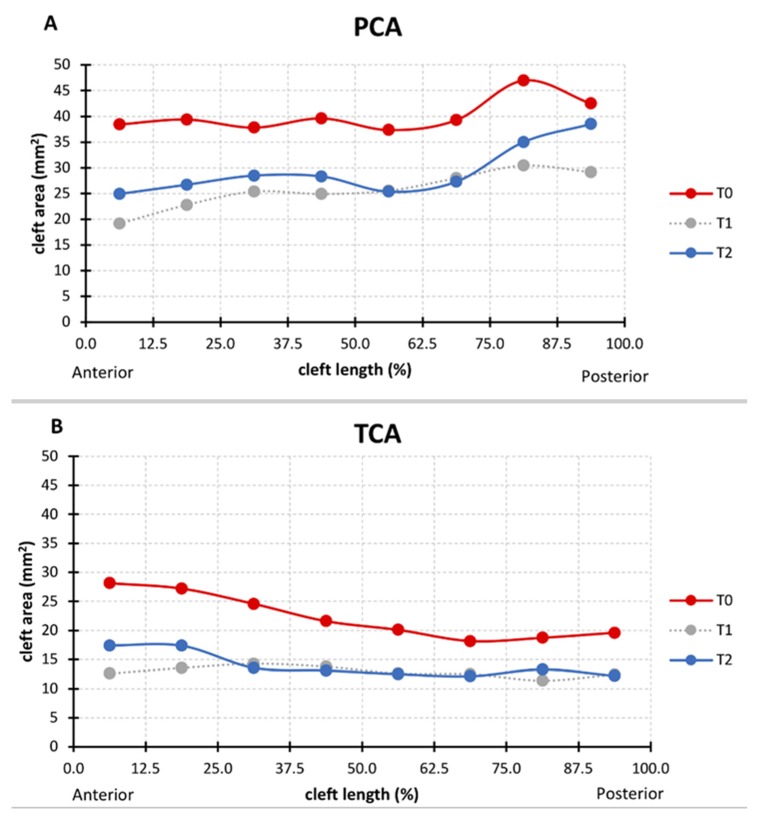
Median (**A**) palatal cleft area (PCA) and (**B**) true cleft area (TCA) at T0, T1, and T2 in eight equidistant quadrangles (0–12.5%, 12.5–25%, 25–37.5%, and so forth) from anterior to posterior.

The median total palatal cleft area (PCA) (GA/GM/GT–LA/LM/LT; see Figure 2A) decreased by 31.5% (from T0 to T2), and the median total true cleft area (TCA) (VA/VM/VT–LA/LM/LT; see Figure 2B) decreased by 34.71% (Table 3). In the anterior section, both PCA and TCA were reduced by around one-third (34.4% and 29.2%, respectively). However, in the middle and posterior sections, the reduction in the cleft area was larger for the true cleft than for the palatal cleft. In the middle section, TCA reduced by 29.2% while PCA reduced by 25.5%. The difference was even more pronounced in the posterior section: 41.3% for TCA compared to 18.3% for PCA (Table 3). The median changes from anterior to posterior of each of the eight equidistant quadrangles of the PCA and TCA are displayed in Figure 5.

### 3.3. Changes in the Height of the Palatal Surface

**Figure 6:**
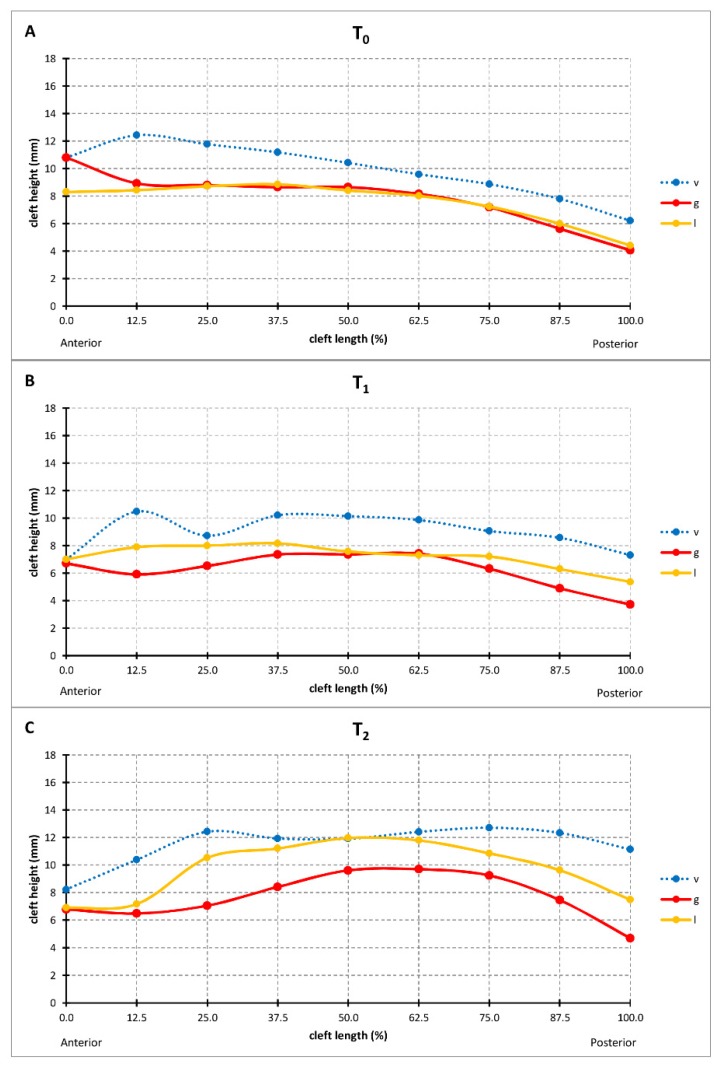
The median vertical height of vomer edge (v height), the junction between the palatal shelf of the greater segment and vomer (g height), and the palatal shelf ridge of the lesser segment (l height) at T0 (**A**), T1 (**B**), and T2 (**C**).

**Figure 7:**
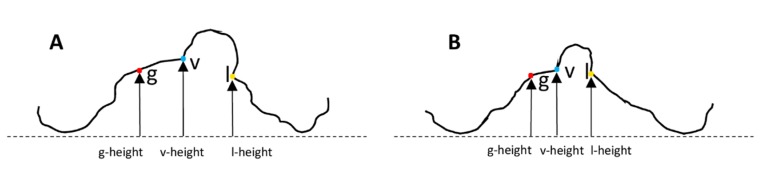
Cross-section through GM and LM perpendicular to the horizontal plane, displaying the height of g, v, and l above the horizontal plane at T0 (**A**) and T2 (**B**).

The height of the palatal surface was measured to the horizontal plane (Q–T/T’) along the longitudinal course of the cleft along three different paths: the greater palatal ridge (g), the lesser palatal ridge (l), and the vomer edge (v) (Figure 2C,D). At birth, the greater and lesser palatal shelf ridges followed a horizontal course at a height of around 8 mm, becoming skewed towards the posterior end. Both shelf ridges run at the same level, while the vomer edge paralleled their course at about 2 mm higher (Figure 6). The heights of the greater and lesser palatal shelf ridges changed from T1 to T2 into a parabolic shape, being highest in the midpalatal section. At (T2), the shelf ridge of the lesser segment still ran parallel to that of the greater segment, but now at 2–3 mm higher. This meant that the shelf ridge of the lesser segment became closer to the course of the vomer edge (Figure 6). The change in the cross-section shape through the midpalatal region (cut through GM and LM perpendicular to the horizontal plane) highlights the change in the height of the palate to the horizontal plane and is displayed in Figure 7.

## 4. Discussion

### 4.1. Three-Dimensional Analysis

The dimensions and shape of the cleft alveolus and palate play an important role in the outcome of any primary surgery [18]. Different methods are used to measure the cleft dimensions, with some investigators only measuring the separation between two segments anteriorly, whereas others measure the cleft width at several palatal levels or measure the cleft area in relation to the total palatal area [2,3,4,5,6,7,10,11,12,13,18,19,20]. Aberrant anatomical structures on dental casts pose a major challenge to the clinician attempting to identify and determine the correct anatomical landmarks [20,21,22]. Vague landmarks are the most important factor contributing to inaccuracy of these measurement, and there is no standard protocol for identifying the points since demarcating these landmarks is extremely difficult [8,12,20,21].

### 4.2. Cleft Width

The palatal cleft width and true cleft width decreased in all cases, which is consistent with previous reports [3,9,18] (Figure 4). The true and palatal cleft width decreased evenly along its longitudinal course, but slightly less in the posterior region, whereas the main effect was already observed at T1 (Figure 4).

A presurgical reduction in the width of the cleft is considered a positive predictor of the surgical results, because this reduces the undermining mobilization of the tissues [5,23]. We reason this is achieved simply by blocking out the depressions on the plaster cast prior to plate fabrication to keep the palatal shelves and vomer surfaces free and not applying any pressure from the tongue. No gradual trimming of the plate was performed, which contrasts with other techniques such as nasoalveolar moulding (NAM) [5] and the Hotz-type plate [24].

### 4.3. Changes in the Palatal and True Cleft Areas

Measuring the cleft area indirectly quantifies the shortage of palatal tissue when performing surgical repairs, and this shortage seems to be related to the amount of subsequent maxillary growth disturbances later in life [5]. Assuming that no tissue is freely transplanted into the cleft area in a primary repair, greater tissue shortage during cleft repair leads inevitably to (a) an increased area of secondary wound healing over soft tissue or bone or (b) an increased volume of dead space between tissues. Both effects are typically present but to variable extents depending on the precise surgical technique used, whether it is tissue turnover, tissue shift in the horizontal or vertical direction, and single- or double-layer tissue closure. However, these two effects are considered to equally increase the invasiveness of surgical repair due to more scarring and a greater risk of wound healing disturbances with, for example fistula formation.

It has been proposed that a given ratio between the cleft area and the entire palatal vault surface within the alveolar ridges can help to define the time point when surgical repair leads to minimal side effects on growth [13]. When considering the effect of surgery on future growth, it is therefore important not only to discriminate between the surgical time point and technique, but also whether the surgical repair addresses the closure of the palatal or (in contrast) the true cleft region only—which constitutes only about half the surface area (Table 3)—and thus markedly reduces the degree of tissue shift.

However, in addition to the total amount of tissue shift differing between PCA and TCA, there are also differences in geometry. This becomes clear when the cleft area is divided into eight sections from anterior to posterior (Figure 5). The palatal cleft area was minimal in the central section while being largest in the posterior section (Figure 5A). Clinical translation of this finding means maximized need for tissue shift in the posterior section of the palate. However, in the posterior section the palatal artery might impede free tissue movement, thus resulting in increased tissue tension, which is also a negative factor for wound healing and thus a risk factor for a residual fistula. Indeed, a meta-analysis [25] identified a fistula as being most frequently located in the posterior hard palate section, at the junction with the soft palate.

In the true cleft region, the area of the posterior section was about the same as that in the central section (Figure 5B). If the surgical cleft closure was limited to the true cleft region, it would have to be investigated whether this could lead to a more homogeneous tissue displacement and tissue tension due to the more even distribution of the area in all sections. 

### 4.4. Changes in the Height of the Palatal Surface

3D changes of the palatal curved surface at different stages have been studied by taking the bilateral tuberosity points, incisal point or canine points, which lacks objectivity and reproducibility [26]. The measurement technique must instead be based on a larger number of data points along a curve denoting the actual height of the palatal segments. Our method offers objectivity, reproducibility, and reliability at different stages (Figure 2C,D). However, the changes measured in the present study cannot be compared to those in previous studies due to differences in the parameters measured and the protocols used when treating cleft lip and palate [27]. With respect to a potential palatal repair after 8 months of pure passive plate therapy, two important discrepancies between TCA and PCA were identified. First, the tissue borders in the true cleft region (the vomer edge and the lesser segment) are vertically closer together than the tissue borders in the palatal cleft region (the greater segment and the lesser segment), thus from a surgical point of view requiring less vertical tissue displacement to come into the same vertical plane for cleft repair, which also minimizes the contiguous amount of dead space (Figure 6C). Second, the tissue borders are higher in the true cleft region than in the palatal cleft region (Figure 7), with this difference being more pronounced in the anterior region (Figure 6C). Thus, a repair following the tissue levels in the true cleft region might allow more space for the tongue in the anterior palatal region to have an undisturbed posture and articulation.

### 4.5. Clinical Translation of the Findings

The long history of presurgical orthopaedic treatment [28] has led to various technical variations depending on the effect aimed for. Some techniques [29] aim to guide the alveolar positions into an optimal position, not mainly for surgery but rather with the intent to optimize the long-term arch form. The plate is periodically trimmed every couple of weeks to guide the alveolar segments, and the orthopaedic therapy continues after lip surgery and soft palate surgery until when hard palate surgery is performed. However, a large randomized controlled trial failed to demonstrate—aside from the narrowing effect before lip surgery [30]—any persistent effect on arch form [31] or occlusion [32] from this specific type of presurgical orthopaedics (Hotz-type plate). Those authors concluded that lip surgery and subsequent palatal surgery overrode the forming effect of presurgical orthopaedics. We therefore refrained from using grinding to actively guide the alveolar segments, instead using a purely passive type of plate therapy similar to the passive appliance of Huddart [28] but without extraoral wires.

In line with the findings of the aforementioned randomized trial, two main ways of clinical reasoning were observed: either presurgical orthopaedics are abandoned and one relies on the palatal shape changes that take place after lip surgery, or the presurgical orthopaedics focus mainly on the narrowing of the anterior cleft region to facilitate the primary repair in the lip, alveolus, and nose region (in NAM), with the palatal shape changes not being taken into account in the primary surgery.

However, the present finding of narrowing of the true cleft region would also allow a third clinical reasoning—using the effect of presurgical orthopaedics in exchange for performing a separate lip repair before palatal closure. As with most one-stage cleft repair techniques, the effect of preoperative orthopaedics is used in exchange for performing a separate lip repair prior to palatal closure. If the results can be confirmed in further studies, the common belief that early isolated lip surgery is necessary to provide optimal conditions for later palatal repair could be questioned.

### 4.6. Strengths and Limitations

We used the vomer edge (also called “Poutriquet’s ridge”) [1,16] and innominate sulcus [16] as new anatomically reproducible landmarks that can be easily used to measure the true cleft width, area, and curvature in all dimensions (Figure 2). To the best of our knowledge, no previous 3D study has differentiated between TCA and PCA. Our measurement errors were found to be well within the ranges found in previous similar studies [8]. The landmarks used in the present study have biological correlates or distinct morphologies that facilitate their identification with high precision and reproducibility [22], which probably explains why the errors in the interrater transverse measurements were less than 0.5 mm, which is lower than that in all previous studies [8,21]. Moreover, no previous study has measured the height of the vomer edge to the horizontal plane. The errors of our interrater measurements were within the acceptable limit of 1.0 mm, making it a practical cleft landmark with a defined cleft anatomical correlate—it is the zenith of the palatal vault prior to surgery (Figure 2D).

The limitation of our study were the small sample size and the lack of a control group without passive plate therapy. We cannot draw any conclusion about whether the passive plate is also purely passive towards the intrinsic growth of each palatal half. Further, no data on a potential long-term effect of the plate therapy on the growth or shape of segments can be provided. This will be further complicated by the additional bias of the wide range of surgical techniques. Future studies involving larger numbers of patients and longer observation times are necessary, as well as 3D evaluations of a control group that does not receive passive plate therapy, in order to fully appreciate the effect of on the growth, remodelling and relocation of the palatal segments and vomer.

## 5. Conclusions

In conclusion, few studies have investigated the morphological changes of the cleft palate width by using 3D standardized, reproducible landmarks in unilateral cleft lip and palate patients. We have introduced the vomer edge for establishing a validated measuring method for the width, area, and height of the true cleft. To the best of our knowledge, this is the first 3D study to show the 3D morphological changes of a pure passive plate therapy over a long period of 8 months in the absence of previous lip surgery. In the investigated cohort without prior lip repair, passive plate therapy provided favourable anatomical conditions for subsequent surgical palatal repair. 

## Figures and Tables

**Table 1:** Definition of landmarks for the 3D analysis.

Abbreviation	Name	Definition
Q	Lateral sulcus vertex	Point where the lateral sulcus intersects the crest of the ridge of the greater segment [4]
T/T’	Tuberosity vertex	Points where the tuberosity border intersects the crest of the ridge of the greater (T) and lesser (T’) segments [10] The base plane runs through T and T’ and is perpendicular to the plane defined by (Q, T, T’) The base line connects T–T’ within the base plane
g	Greater ridge	Path of the greater segment’s palatal shelf ridge, that is at the junction with the vomer [1]
v	Vomer edge	Path along the maximal curvature of the vomer
l	Lesser ridge	Path of the lesser segment’s palatal shelf ridge
GA (=VA)	Greater anterior (=Vomer anterior)	Most-anterior point on the ridge of the greater segment where it intersects with the vomer edge
GT	Greater posterior	Point where the ridge of the greater segment intersects the base plane
GM	Greater midpoint	Point halfway between GA and GT following the path on the ridge of the greater segment
LA	Lesser anterior	Most-anterior point on the ridge of the lesser segment ridge
LT	Lesser posterior	Point where the ridge of the lesser segment intersects the base plane
LM	Lesser midpoint	Point halfway between LA and LT following the path on the ridge of the lesser segment
VT	Vomer posterior	Point where the vomer edge intersects the base plane
VM	Vomer midpoint	Point halfway between VA (=GA) and VT following the path on the vomer edge

**Table 2:** Definitions of 3D landmark measurements.

Abbreviation	Description
Cleft area dimensions	
GA/GM/GT–LA/LM/LT	Total palatal cleft area (PCA)
VA/VM/VT–LA/LM/LT	Total true cleft area (TCA)
Transverse dimensions	
GA–LA	Anterior palatal cleft width
GM–LM	Middle palatal cleft width
GT–LT	Posterior palatal cleft width
VA–LA	Anterior true cleft width
VM–LM	Middle true cleft width
VT–LT	Posterior true cleft width
Vertical dimensions^1^	
g-height	Height of the palatal shelf ridge of the greater segment perpendicular to the horizontal plane
l-height	Height of the palatal shelf ridge of the lesser segment perpendicular to the horizontal plane
v-height	Height of the vomer edge perpendicular to the horizontal plane

^1^ Vertical dimension measured at nine equidistant points along the paths g, l, and v.

**Table 3:** Measured changes in palatal cleft area (PCA) and true cleft area (TCA).

Cleft Area (mm^2^)	Section		T0 Median (IQR)		T2 Median (IQR)		*p*-Value
PCA	Total	0%–100%	334	(294.9–349.8)	228.8	(205–287.9)	0.0015
	Anterior	0–25%	75.3	(67.2–93.3)	49.4	(32.0–70.0)	0.0090
	Middle	25–75%	157.0	(141.5–173.8)	116.9	(99.7–135.0)	0.0076
	Posterior	75–100%	91.8	(77.5–102.6)	75.0	(61.5–84.2)	0.0090
TCA	Total	0–100%	185.4	(151.5–220.1)	121.1	(100.2–144.6)	0.0015
	Anterior	0–25%	56.9	(40.9–66.6)	41.7	(18.1–51.3)	0.0409
	Middle	25–75%	84.7	(69.6–102.8)	60.0	(42.3–62.2)	0.0007
	Posterior	75–100%	41.2	(31.5–48.5)	24.2	(20.3–32.35)	0.0012

PCA, palatal cleft area; TCA, true cleft area; IQR, interquartile range.
